# Low dispersion in the infectiousness of COVID-19 cases implies difficulty in control

**DOI:** 10.1186/s12889-020-09624-2

**Published:** 2020-10-16

**Authors:** Daihai He, Shi Zhao, Xiaoke Xu, Qiangying Lin, Zian Zhuang, Peihua Cao, Maggie H. Wang, Yijun Lou, Li Xiao, Ye Wu, Lin Yang

**Affiliations:** 1 Department of Applied Mathematics, Hong Kong Polytechnic University, Hong Kong, China; 2 JC School of Public Health and Primary Care, Chinese University of Hong Kong, Hong Kong, China; 3 CUHK Shenzhen Research Institute, Shenzhen, China; 4 College of Information and Communication Engineering, Dalian Minzu University, Dalian, 116600 China; 5 Michigan Institute for Data Science at University of Michigan, Ann Arbor, MI USA; 6 Clinical Research Center, Zhujiang Hospital, Southern Medical University, Guangzhou, China; 7 College of Medical Information Engineering, Chengdu University of Traditional Chinese Medicine, Chengdu, China; 8 Computational Communication Research Center, Beijing Normal University, Zhuhai, 519087 China; 9 School of Journalism and Communication, Beijing Normal University, Beijing, 100875 China; 10 School of Nursing, Hong Kong Polytechnic University, Hong Kong, China

**Keywords:** COVID-19, Basic reproductive number, Dispersion, Negative binomial, Mitigation

## Abstract

The individual infectiousness of coronavirus disease 2019 (COVID-19), quantified by the number of secondary cases of a typical index case, is conventionally modelled by a negative-binomial (NB) distribution. Based on patient data of 9120 confirmed cases in China, we calculated the variation of the individual infectiousness, i.e., the dispersion parameter *k* of the NB distribution, at 0.70 (95% confidence interval: 0.59, 0.98). This suggests that the dispersion in the individual infectiousness is probably low, thus COVID-19 infection is relatively easy to sustain in the population and more challenging to control. Instead of focusing on the much fewer super spreading events, we also need to focus on almost every case to effectively reduce transmission.

## Introduction

Since the early outbreak of coronavirus disease 2019 (COVID-19) pandemic, huge efforts have been devoted on estimating key epidemiological parameters due to their important implication in mitigation planning. For instance, according to a survey posted in a public domain (https://github.com/midas-network/COVID-19/tree/master/parameter_estimates/2019_novel_coronavirus), there were at least 47 studies (either peer-reviewed or not) on the cumulative case count in a location have been posted, 39 works on the reproductive number *R*_0_ (number of secondary cases may be cause by a typical primary cases), 13 on the incubation period (time delay between infection and symptom onset), 6 on the serial interval or generation interval (time delay between symptom onset or infection of an index case and its secondary case in a transmission chain), 6 on the symptomatic case fatality ratio. However, the individual variation in infectiousness, the dispersion rate (*k*), has been largely overlooked, except for one early work in Eurosurveillance [1]. He et al. (2020) summarized the recent estimates on *k* from empirical offspring distributions, including 0.58 (95% confidence interval [CI]: 0.35, 1.18) of Bi et al. (2020) from a sample of 391 COVID-19 cases in Shenzhen China [2]. It is of note that there is mathematical modelling work based on imported and reported case numbers in a variety of countries showing that *k* could be 0.1 (95% CI: 0.05, 0.2) [3]. The recent study of Lau et al. [4] used a spatiotemporal transmission process model and estimated that overall dispersion parameter *k* is 0.45 for Cobb County, 0.43 for Dekalb, 0.39 for Fulton, 0.49 for Gwinnett, and 0.32 for Dougherty in Georgia, USA. In this work, with a larger dataset, we calculate *k* using the empirical offspring distribution approach. Our data are from mainland China where strict surveillance guaranteed the quality of the data. Since we adopted the basic definition approach, our methods do not rely on additional assumptions typically needed for mathematical modelling.

## Method

Negative binomial distribution (NB) is used to model the distribution of secondary case numbers, i.e., the offspring numbers, of an index case. The dispersion parameter, *k*, (i.e., size, which is nonnegative) controls the variation of the NB distribution. A sufficiently small *k* implies that the majority of disease transmission was driven by a few super-spreaders, and thus the spread is likely to be controlled by preventing super-spreading events. A large *k* implies that the NB distribution approaches a Poisson distribution, and the virus easily persist and is difficult to eradicate. Following the pioneer work of Lloyd-Smith et al. [5], we assume that the number of secondary cases, denoted by *Z*, for a typical primary case, follows NB (mean = *R*_0_, dispersion = *k*), and thus the variance is *R*_0_ + *R*_0_^2^/*k*. When *k* is sufficiently small, the distribution will have a peak at 0, and in the limit when *k* = 0, the NB distribution is concentrated at zero. When *k* = 1, the distribution is a geometric distribution; and when *k* approaches infinity, the NB distribution approaches a Poisson distribution with both mean and variance equal to *R*_0_ [5].

## Results and discussion

The *k* plays an important role in explaining the wide spreading of COVID worldwide, given a similar *R*_0_ as the other coronavirus, i.e., the severe acute respiratory syndrome (SARS). Lloyd-Smith et al. [5] estimated a smaller *k* = 0.16 for the SARS outbreak in Singapore in 2003.

**Fig. 1:**
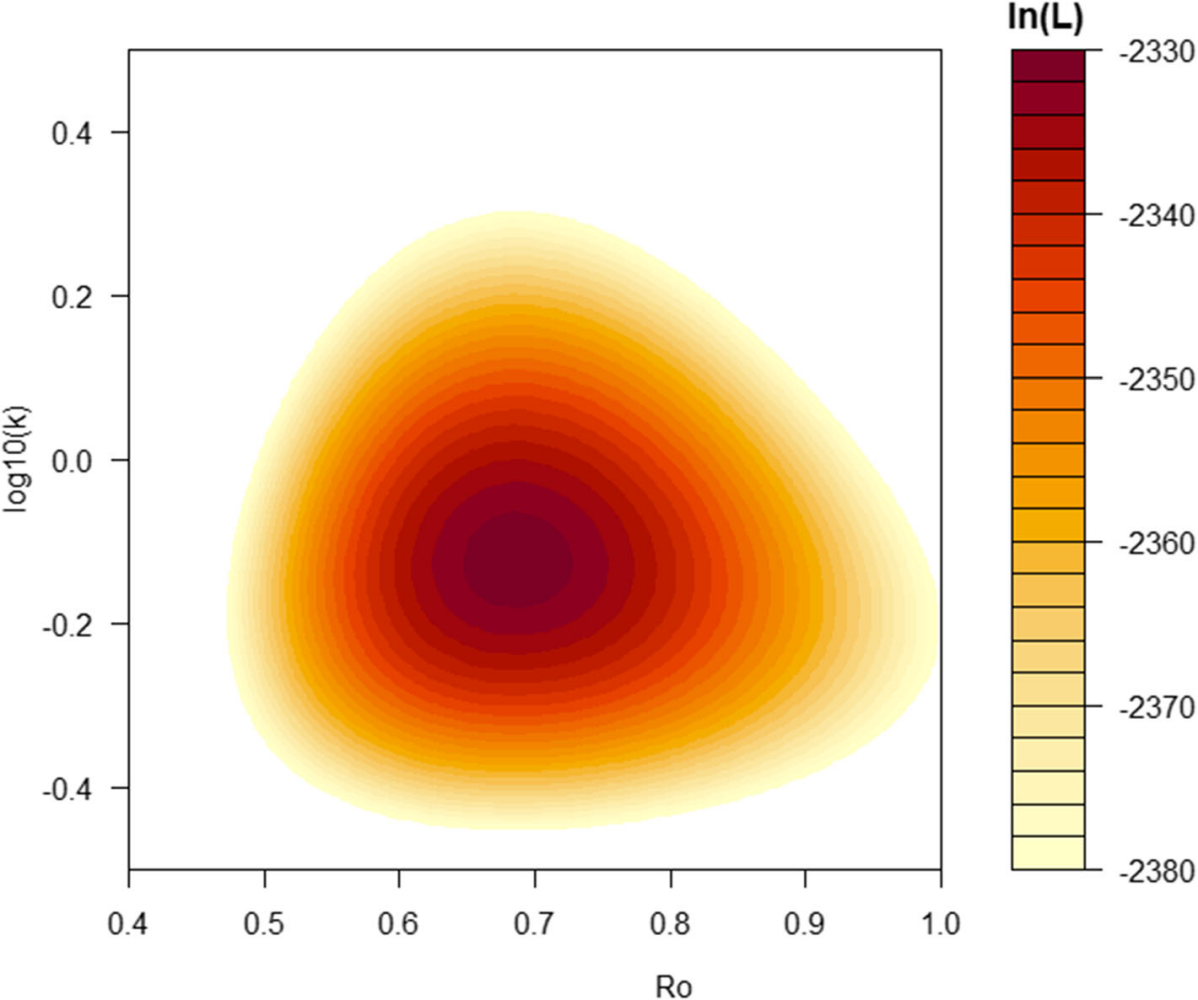
Profile Log likelihood of the NB model given the data in *R*_0_ versus *k* plane based on data listed in Table 2

We first tried Riou et al.’s [1] method to calculate the *R*_0_ and *k* in six countries (see Table 1), and found that *R*_0_ is in line with World Health Organization (WHO) early estimates, while *k* cannot be reliably estimated. Then we obtained the numbers of secondary cases from a study by Xu et al. [9] (see Table 2), and estimated *k =* 0.7 (95%CI 0.59, 0.98) and *R*_0_ = 0.69 (95%CI: 0.62, 0.77) using profile likelihood approach and the profile Log likelihood of the NB model given the data in *R*_0_ versus *k* plane is shown in Fig 1. This estimate is larger than that of SARS around 0.16, but close to that of the 1918 pandemic influenza 0.94 (95%CI 0.59, 1.72) [2]. Our estimate is in line with Bi et al. 0.58 with 95% CI: 0.35, 1.18) [10]. However, we have 9120 confirmed cases, compared to Bi et al. 391 confirmed cases, and thus our estimate has a smaller confidence interval.

**Table 1:** Summary of estimated *R*_0_ of COVID-19 outbreaks in six countries

Country	Time interval	***R***_**0**_
**France**	20/2/2020–12/3/2020	3.5 (3.2, 3.8)
	7/2/2020–12/3/2020	2.0 (1.7, 2.3)
**UK**	21/2/2020–12/3/2020	2.9 (2.6, 3.2)
	11/2/2020–12/3/2020	2.0 (1.7, 2.3)
**Singapore**	23/1/2020–23/2/2020	1.7 (1.4, 2.0)
**Germany**	21/2/2020–12/3/2020	3.5 (3.2, 3.8)
	11/2/2020–12/3/2020	2.3 (2.0, 2.6)
**Spain**	21/2/2020–12/3/2020	3.5 (3.2, 3.8)
	11/2/2020–12/3/2020	2.3 (2.0, 2.6)
**Japan**	23/1/2020–12/3/2020	1.7 (1.4, 2.0)
	11/2/2020–12/3/2020	2.3 (2.0, 2.6)

We adopted a similar method as in [1], and simulate a Negative-binomial process to match the observed daily cases in these country over the chosen time period when the number grew exponentially. Using a maximum likelihood approach to infer *R*_0_. The method is also explained in [6–8]

**Table 2:** Frequency of primary cases as a function of the numbers of secondary cases per primary case. Data are from [9]

Numbers of secondary cases	Frequency of primary cases
0	1241
1	511
2	160
3	71
4	33
5	15
6	7
7	3
8	2
9	3
10	1
11	1
12	1
17	1

Our results suggest that the majority of the COVID-19 transmission is not due to super-spreading events. The number of secondary cases of a primary case roughly follows a geometric distribution, large proportion of primary cases have potentials to generate more than one secondary cases. This indicates that COVID-19 is easy to persist in the general population if strong measure is not taken, given the similar *R*_0_ as SARS. Therefore, outbreak mitigation is relatively difficulty without taking extreme efforts such as city lockdown.

## Data Availability

Data used to calculate the *R*_0_ for six countries are publicly available at https://covid19.who.int/table. Data used to calculate the *k* for China are given in Table 2.

## Abbreviations

CI: Confidence interval
COVID-19: Coronavirus disease 2019
NB: Negative binomial distribution
SARS: Severe acute respiratory syndrome
